# Switching to Pitavastatin in Statin-Treated Low HDL-C Patients Further Improves the Lipid Profile and Attenuates Minute Myocardial Damage

**DOI:** 10.4021/jocmr1108w

**Published:** 2012-11-11

**Authors:** Chikao Ibuki, Yoshihiko Seino, Toshiaki Otsuka, Nakahisa Kimata, Toru Inami, Ryo Munakata, Kyoichi Mizuno

**Affiliations:** a Cardiovascular Center, Nippon Medical School Chiba Hokusoh Hospital, Chiba, Japan; b Department of Hygiene and Public Health, Nippon Medical School, Tokyo, Japan; c Division of Cardiology, Nippon Medical School, Tokyo, Japan

**Keywords:** Statin, High-density lipoprotein-cholesterol, Minute myocardial damage, Troponin

## Abstract

**Background:**

The aim of this study is to determine the prevalence of minute myocardial damage (MMD) in already statin-treated dyslipidemic patients with a low high-density lipoprotein-cholesterol (HDL-C) level, and to evaluate whether pitavastatin could affect the lipid profiles and biomarkers reflecting myocardial stress and injury.

**Methods:**

Twenty patients (15 men; age 66 ± 8) being treated with any statin but who had HDL-C < 40 mg/dL, were switched to pitavastatin (2 mg/day) treatment. The patient lipid profiles and the levels of N-terminal pro-brain natriuretic peptide (NT-proBNP), high-sensitive troponin T (hsTnT), and high-sensitive C-reactive protein (hs-CRP) were evaluated for six months.

**Results:**

At three months after the statin replacement, the HDL-C significantly increased from 37 ± 3 mg/dL to 40 ± 5 mg/dL (P < 0.05), and the low-density lipoprotein-cholesterol (LDL-C) and LDL-C/HDL-C ratio significantly reduced (100 ± 28 mg/dL to 86 ± 22 mg/dL, P < 0.05; 2.68 ± 0.67 to 2.17 ± 0.64, P < 0.05, respectively), and these changes were sustained for six months. In the whole study population, no significant changes were observed in the NT-proBNP, hsTnT, or hsCRP for six months. However, in 11 cases who showed a positive (> 0.003 ng/mL) hsTnT at baseline, a significant reduction in the hsTnT was observed (0.016 ± 0.020 ng/mL to 0.014 ± 0.020 ng/mL, P < 0.05), and its percent reduction significantly correlated with the percent increase in HDL-C (r = -0.68, P < 0.05).

**Conclusions:**

MMD (positive hsTnT) was observed in more than half of patients with low HDL-C despite the administration of any statin, and the replacement of their previous statin with pitavastatin further improved their lipid profiles and led to better myocardial protection, possibly mediated via the elevation of the HDL-C level.

## Introduction

High-density lipoprotein cholesterol (HDL-C) has an atheroprotective effect in blood vessels [1, 2], and it has been established that a low serum level of HDL-C is a cardiovascular risk factor independent from the low-density lipoprotein cholesterol (LDL-C) level [3]. As clinical trials have consistently shown that the pharmacological intervention using inhibitors of 3-hydroxy-3-methylglutaryl coenzyme A reductase (statins) result in favorable effects on cardiovascular morbidity and mortality in patients with hypercholesterolemia and/or cardiovascular diseases [4-7], lowering the LDL-C has been the mainstay of therapy for dyslipidemic patients. In those trials, however, substantial elevation of the HDL-C level (5-15%) has also been reported, and raising the HDL-C level may contribute, at least in part, to the benefit of statins. In fact, it has been demonstrated in a meta-analysis that a decrease in LDL-C and an increase in HDL-C are independent predictors of coronary atheroma regression [8].

Statin therapy has been shown to exhibit myocardial protection when it is used for patients undergoing percutaneous coronary intervention [9]. Minute myocardial damage (MMD), that can become detectable with cardiac sensitive and specific biomarkers such as troponins, has been documented in patients with heart failure and stable coronary artery disease, and the serum troponin levels are associated with the disease severity and prognosis [10, 11]. Moreover, the development of a high sensitive assay of troponin T has made it possible to detect MMD even in subjects without apparent cardiovascular disease, and the pathogenic contribution of coronary risk factors in MMD has been proposed [12]. However, it remains unknown how dyslipidemia, particularly that complicated with a low level of HDL-C, indicates MMD, and whether the MMD observed in such patients can be influenced by statin therapy.

We performed the present study to determine the prevalence of MMD in already statin-treated dyslipidemic patients with a low HDL-C level, and to evaluate whether pitavastatin, which has been shown to most prominently increase the serum HDL-C among the various statins [13], could affect both the lipid profile and biomarkers indicating myocardial stress and injury in these patients.

## Materials and Methods

### Study population

This study was performed from October 2010 to August 2011 at the outpatient clinic of the Cardiovascular Center, Nippon Medical School Chiba Hokusoh Hospital. Dyslipidemic patients with a HDL-C value below 40 mg/dL despite the prescription of any statin for at least six months, with an age of 20 - 80 years old, were enrolled in this study. The exclusion criteria were acute coronary syndrome or coronary recanalization therapy within six months, impaired renal (serum creatinine ≥ 1.5 mg/dL) and/or liver function, heart failure, pregnancy, familial hypercholesterolemia or a terminal systemic condition of any etiology. This study was approved by the Investigation Review Board of the hospital, and all patients gave written informed consent for their participation.

### Protocol

In all patients, the statin was changed to pitavastatin (2 mg/day). The lipid profile and standard biochemical parameters were measured at baseline, and again at three and six months after initiating the treatment with pitavastatin. The levels of N-terminal pro-brain natriuretic peptide (NT-proBNP), high-sensitive troponin T (hsTnT), and high-sensitive C-reactive ptotein (hsCRP) were assessed at baseline and six months.

### Biochemical measurements

Blood samples were obtained intravenously after overnight fasting. Standard enzymatic methods were adopted for measurement of the serum total cholesterol (TC), triglycerides (TG), and creatinine. The serum HDL-C level was measured using the direct method. The LDL-C level was determined using Friedwald’s equation as follows, unless the TG level was below 400 mg/dL [14]: LDL-C = TC - (HDL-C + TG/5).

The serum NT-proBNP and hsTnT were measured using an electrochemiluminescence immunoassay (Roche Diagnostics), and hsCRP was measured by a latex turbidimetric immunoassay (Siemens Healthcare Diagnostics). The lower detection limit of the hsTnT assay was 0.003 ng/mL. Other biochemical parameters were measured with the UV method using an autoanalyzer (Hitachi 7,700DPP). The estimated glomerular filtration rate (eGFR) was calculated according to the following equation presented by the Japanese Society of Nephrology [15]: eGFR (mL/min/1.73m^2^) = 194 × serum creatinine^-1.094^ × age^-0.287^ (× 0.739, if female).

### Statistical analysis

Numerical values were expressed as the means ± standard deviation. Serial changes in the lipid profile were assessed with a repeated analysis of variance followed by Dunnet’s t-test. Changes in the NT-proBNP, hsTnT, and hsCRP levels were assessed with a Wicoxon t-test. The correlation between lipid profiles and the hsTnT level was analyzed with Spearman’s test. Values of P < 0.05 were regarded as being statistically significant.

## Results

The baseline characteristics of the study subjects are shown in Table 1. The mean age was 66.4 years and 75% of subjects were male. The most prevalent co-morbidity was hypertension, and more than half patients had been administered antihypertensives such as angiotensin receptor blockers and calcium channel blockers. In 90% of the patients, the statin being administered before the study enrollment was pravastatin or atorvastatin.

**Table 1:** Baseline Patient Characteristics

Age, years	66.4 ± 7.8
Gender (%)	Male, 15 (75%); Female, 5 (25%)
Body mass index	24.8 ± 3.4
Current smokers (%)	5 (25%)
Co-morbidity (%)	HT 11 (55%), DM 6 (30%), CAD 8 (40%)
Statin before the enrollment (%)	Pravastatin 10 (50%); Simvastatin 1 (5%); Atorvastatin 8 (40%); Rosuvastatin 1 (5%)
Co-administration (%)	CCB, 13 (65%); ACEI and/or ARB, 12 (60%); BB, 8 (40%); Diuretics, 3 (15%), Anti-thrombotics, 10 (50%)

HT: hypertension, DM: diabetes mellitus; CAD: coronary artery disease; CCB: calcium channel blocker; ACEI: angiotensin converting enzyme inhibitor; ARB: angiotensin receptor blocker, BB: beta blocker.

At three months after the replacement of the statin with 2 mg/day pitavastatin, the TC and LDL-C were significantly reduced (-9.1% and -14.0%, respectively), and the HDL-C level was significantly elevated (+8.1%), resulting in a significant reduction in the LDL-C/HDL-C (L/H) ratio (Table 2). These improvements in the lipid profile were sustained for another three months. Neither complications nor abnormal laboratory fluctuations were shown noted in the present study patients after the statin replacement.

**Table 2:** Serial Changes in the Laboratory Data of the Overall Subjects

	Baseline	3 months	6 months
TC (mg/dL)	176 ± 29	160 ± 27*	167 ± 24
HDL-C (mg/dL)	37 ± 3	40 ± 5*	41 ± 6*
TG (mg/dL)	192 ± 80	169 ± 78	187 ± 69
LDL-C (mg/dL)	100 ± 28	86 ± 22*	89 ± 24*
L/H ratio	2.68 ± 0.67	2.17 ± 0.64**	2.18 ± 0.68*
Creatinine (mg/dL)	0.89 ± 0.20	0.88 ± 0.21	0.90 ± 0.22
AST (IU/L)	24 ± 9	26 ± 9	26 ± 12
ALT (IU/L)	26 ± 13	27 ± 11	27 ± 12
CPK (IU/L)	125 ± 173	102 ± 54	91 ± 38
NT-proBNP (pg/mL)	181 ± 272		257 ± 440
hsTnT (ng/mL)	0.009 ± 0.011		0.007 ± 0.009
hsCRP (ng/mL)	2,994 ± 6,572		884 ± 653

TC: total cholesterol; HDL-C: high density lipoprotein cholesterol; TG: triglycerides; LDL-C: low density lipoprotein cholesterol; L/H: LDL-C/HDL-C; AST: asparatate aminotransferase; ALT: alanine aminotransferase; CPK: creatine phosphokinase; NT-pro BNP: N-terminal pro-brain natriuretic peptide; hsTnT: high sensitive troponin T; hsCRP: high sensitive C-reactive protein. ^*^: P < 0.05 vs baseline, ^**^: P < 0.01 vs baseline

**Figure 1:**
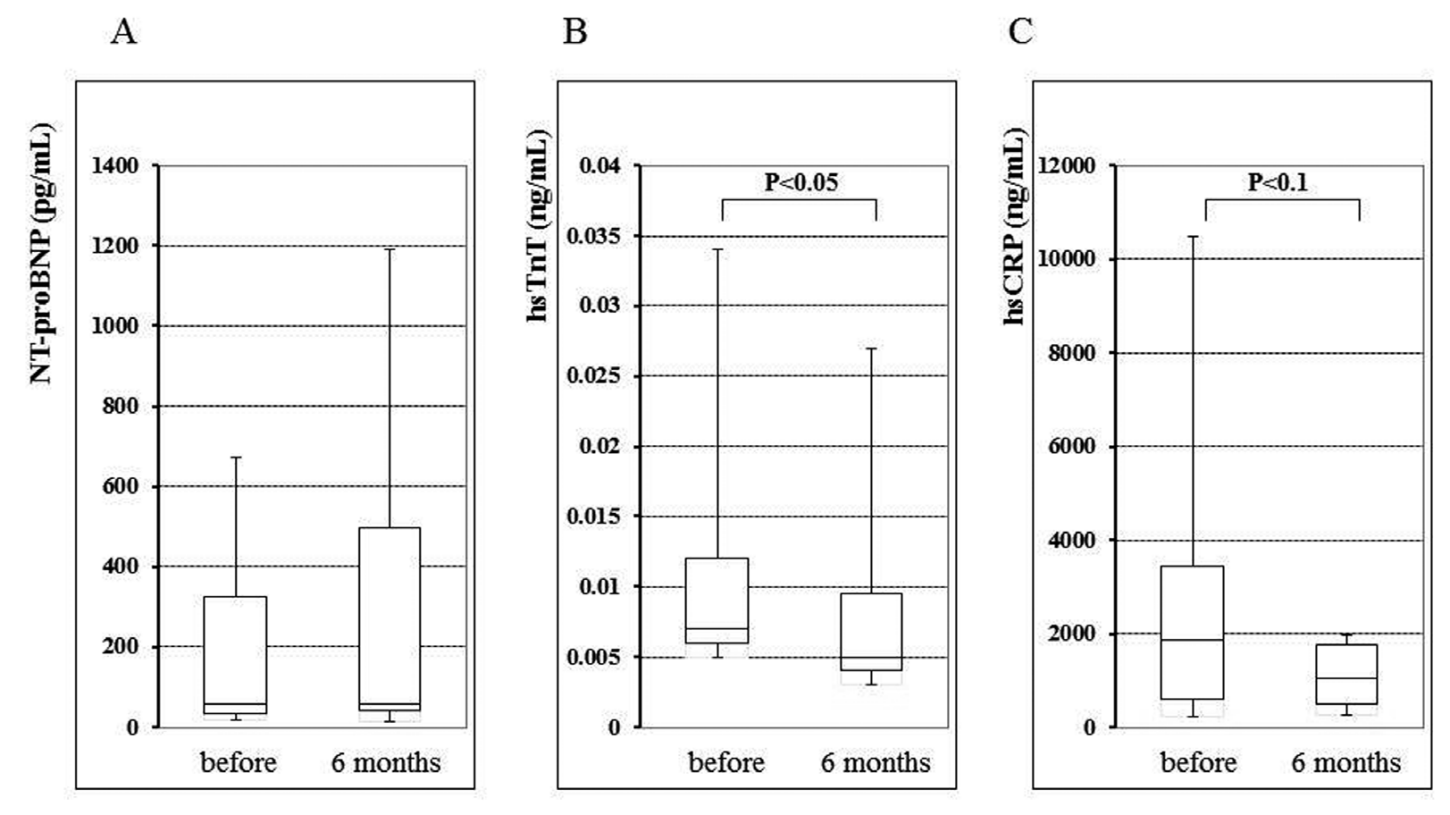
Serum N-terminal pro-brain natriuretic peptide (NT-proBNP) (A), high-sensitive troponin T (hsTnT) (B), and high-sensitve C-reactive protein (hsCRP) (C) levels before and at six months after the replacement of the previous statin with 2 mg/day pitavastatin.

**Figure 2:**
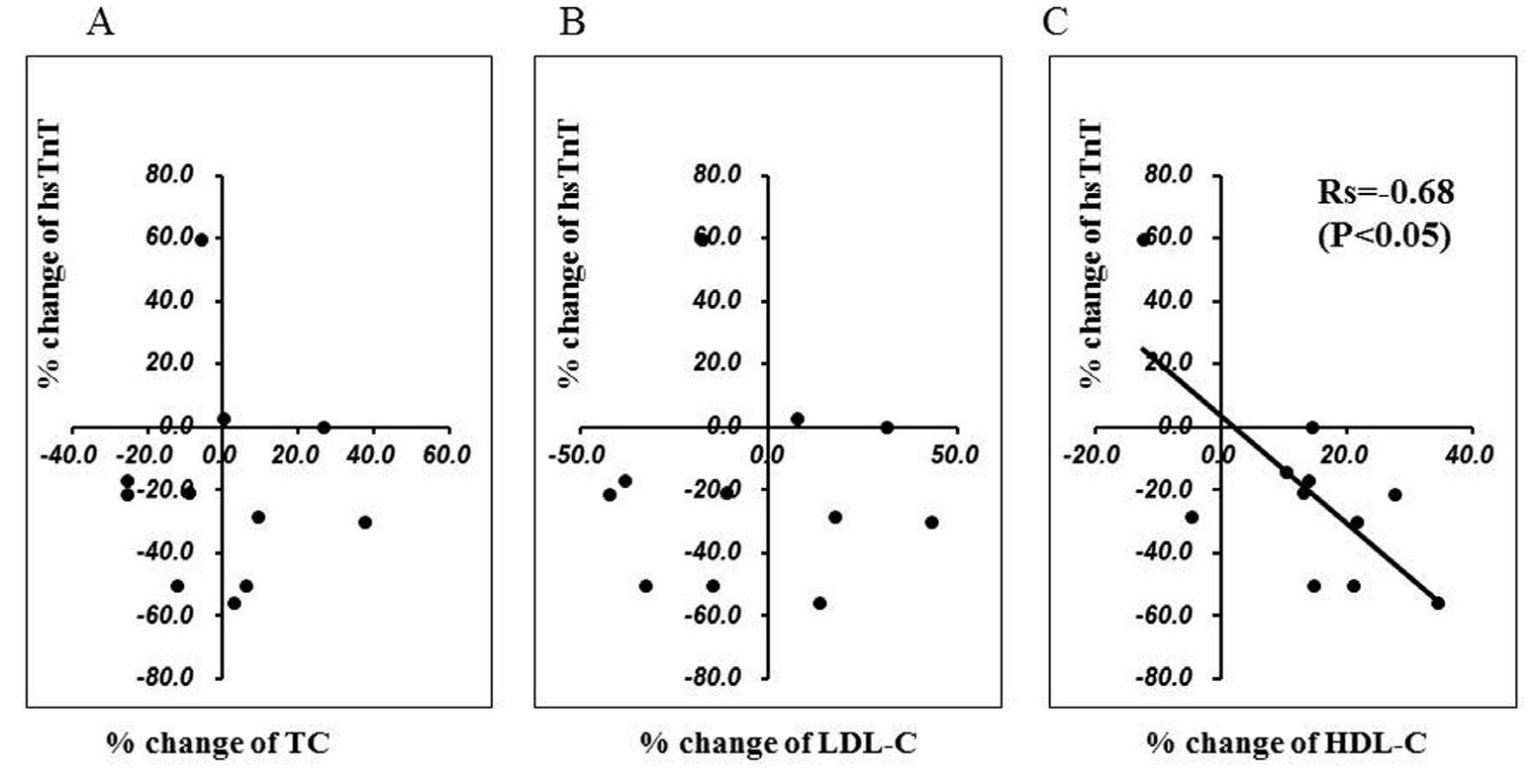
The relationship between the percent change in the high-sensitive troponin T (hsTnT) level (vertical axis) and the percent change in the total cholesterol (TC) (A), low-density lipoprotein cholesterol (LDL-C) (B), and high-density lipoprotein cholesterol (HDL-C) (C) levels (horizontal axis, respectively) for six months after the statin replacement. The percent change in the hsTnT inversely correlated with the change in the HDL-C level (Rs = -0.68, P < 0.05).

In the entire study population, no significant changes were observed in the NT-proBNP, hsTnT, or hsCRP for six months after the commencement of pitavastatin treatment. There were no statistically significant differences in these biomarkers at baseline between CAD and non-CAD patients (0.009 ± 0.011 ng/mL and 0.011 ± 0.009 ng/mL, respectively, NS). However, at baseline, 55% of patients showed a positive (> 0.003 ng/mL) hsTnT value, and a statistically significant reduction in the hsTnT was observed in such patients at the six month evaluation (baseline 0.016 ± 0.020 ng/mL; 6 months 0.014 ± 0.020 ng/mL, P < 0.05) (Fig. 1), although in the patients without a positive hsTnT value, there was not significant change during this period (baseline 0.003 ± 0.001 ng/mL; 6 months 0.003 ± 0.001 ng/mL). In positive hsTnT patients, the percent change of hsTnT during the six months significantly correlated with the percent change in the HDL-C value (r = -0.68, P < 0.05) among the various lipid parameters (Fig. 2). There was no significant difference in the hsTnT at baseline between the CAD and non-CAD patients (0.009 ± 0.011 ng/mL and 0.011 ± 0.009 ng/mL, respectively, NS).

## Discussion

In the present study, the replacement of another statin with pitavastatin in dyslipidemic patients with a low HDL-C level resulted in a further improvement in the lipid profile for three to six months without deleterious effects. Although patients with acute coronary syndrome or recent coronary recanalization therapy were excluded in this study, more than half of the patients showed a positive (> 0.003 ng/mL) hsTnT value, and in these patients, a significant reduction in the hsTnT level was observed at six months after the statin replacement. Interestingly, such a reduction in the hsTnT significantly correlated with the increase in the HDL-C, but not with changes in other lipid parameters.

### Improvement of the lipid profile by pitavastatin

It has been shown in epidemiological studies that a low level of HDL-C is a potential risk factor for future cardiovascular events, independent of the LDL-C level [16-18], and that an increase in the HDL-C level is associated with a reduced risk of coronary artery disease[19, 20]. Regarding the clinical significance of the elevation of HDL-C induced by a pharmacological intervention, Nicholls et al have demonstrated, in a meta-analysis of four controlled trials using intra-vascular ultrasound, that substantial cononary plaque regression was observed in patients who had a LDL-C of less than 87.5 mg/dL or a percent increase in the HDL-C of more than 7.5% after statin treatment [8]. This finding suggests that statin-induced elevation of the HDL-C level is associated with anti-atherosclerotic effects in the cardiovascular system, although it is still unclear as to whether the atherosclerotic regression, or at least the slowing of progression, can translate to a meaningful improvement in the clinical outcome.

Pitavastatin is characterized as a statin that has a potent action to increase the HDL-C level. The LIVES study [21], a prospective post-marketing surveillance study in which 20,000 Japanese dyslipidemic patients were enrolled, showed an apparent reduction of LDL-C by 31.3% and an increase of HDL-C by 5.9% with the administration of 2 mg of pitavastatin daily. In that study, the favorable effect of pitavastatin on the HDL-C level was more prominent in the low HDL-C subgroup (24.6% increases in the HDL-C concentration). Although the increase in the HDL-C level by 8.1% shown in the present study is minor compared to the finding observed in the low HDL-C subgroup of the LIVES study, it should be noted that in our study, pitavastatin was administered to patients who had already been previously treated with other statins, and therefore, not to statin-naive patients.

The mechanism(s) by which statins increase the HDL-C level remains unknown. Possible mechanisms include remodeling of HDL-C particles [22], inhibition of the activity of the cholesterol-ester transfer protein (CETP) [23], and statin-induced promotion of hepatic apolipoprotein A-I production [24]. Pitavastatin has been shown to promote the expression of apolipoprotein A-I and ABCA1 mRNA more efficiently than other statins [25]. Although pitavastatin has been shown to have as potent an anti-atherosclerotic action as atorvastatin in patients with acute coronary syndrome in the JAPAN-ACS study [26], it is unclear whether such beneficial effects are attributable to the increase in the HDL-C level or to the direct action of pitavastatin.

### Attenuation of myocardial injury

Cardiac troponins are sensitive and specific biomarkers of myocardial injury, and are the prerequisite tool for the diagnosis of acute coronary syndrome [27-29]. It has been reported that many patients with heart failure show an elevation of the serum troponin T level, and its elevation is a predictive factor of the disease outcome [30-33], suggesting a profound impact for ongoing minute myocardial damage (MMD) in the pathogenesis of heart failure. With regard to the prevalence of positive troponin T in stable coronary artery disease patients, Omland et al, using a high sensitive assay for troponin T which was 10 times more sensitive than the conventional assays, have demonstrated that 97% of patient show hsTnT values above the detection limit (0.001 ng/mL) [11]. We have previously reported that, in a work-based population free from cardiovascular disease, 80% of subjects show positive (> 0.002 ng/mL) hsTnT values, and that the levels are associated with the Framingham risk prediction score [12]. The present data showing that more than half of patients have signs of MMD at baseline despite statin therapy is consistent with the previous findings, and suggests that dyslipidemia can result in myocardial injury even in the absence of heart failure or acute coronary syndrome.

Statins are known to alleviate peri-procedural myocardial injury in patients undergoing coronary intervention, partly due to inhibition of coronary microembolism resulting from the plaque rupture [9]. The mechanism underlying the attenuation of MMD observed after the statin replacement with pitavastatin cannot be clarified from the present data. However, this is the first paper that evaluated the effect of pitavastatin on the hsTnT level in dyslipidemic patients already being treated with another statin. The significant correlation between the percent change in the HDL-C level and the reduction of the hsTnT observed in the subpopulation of patients who were hsTnT positive in the present study may mean that HDL-C particles could prevent myocardial injury via coronary plaque stabilization. In this regard, although 40% of our patients had an apparent history of stable CAD, a substantial number of non-CAD patients may also have suffered from silent or undiagnosed CAD. In fact, we have reported that vulnerable plaques can exist in angiographically non-diseased coronary arteries [34]. Therefore, there is a possibility that the increase in the HDL-C level induced by pitavastatin stabilized coronary silent vulnerable plaques in both CAD and non-CAD patients.

However, it has not been explicitly established that the quantitative change *per se* in the serum HDL-C level results in anti-atherosclerotic effects. Clinical trials adopting pharmacological inhibitors of CETP have hitherto failed to establish any clinical benefit, despite their substantial elevation of the HDL-C level [35]. It is possible that pitavastatin might have exerted its myocardial protection through mechanisms independent from those related to lipid metabolism. For example, it is possible that the anti-inflammatory action of pitavastatin contributed to the protection in the present study [36], as hsCRP tended to decrease after pitavastatin treatment in the subpopulation showing MMD.

### Limitations of the study

The present study has several limitations. First, this study enrolled a small number of patients with an open and single arm design. However, the present study was a proof-of-concept study performed to investigate the effects of statin replacement with pitavastatin on the lipid profile and MMD for dyslipidemic patients who had a low HDL-C level despite treatment with other statins. Whether the elevation of the HDL-C by the statin replacement results in beneficial outcomes warrants a large scale study in the future. Second, the present study included both CAD and non-CAD patients. In general, these 2 types of patients have quite different backgrounds in terms of their future risk of cardiovascular events, and therefore, the achievement target of LDL-C level is low in patients with CAD (< 100 mg/dL) for the secondary prevention according to the guidelines of the Japanese Atherosclerosis Society [37]. The target HDL-C level is, however, the same (≥ 40 mg/dL) regardless of the presence of CAD, and the use of strict statin therapy is rational for both types of patients.

### Conclusions

The replacement of other statins with pitavastatin in dyslipidemic patients with a low HDL-C resulted in further improvement in the lipid profile for at least six months. Furthermore, the statin replacement decreased the hsTnT value during this period in patients with a positive hsTnT value at baseline, and its reduction significantly correlated with an increase in the HDL-C. In dyslipidemic patients with a low HDL-C level, pitavastatin should be administered for better management of the lipid profile and possible attenuation of MMD.
